# Stability Prediction for UGV Based on Drone-Borne LiDAR Terrain Mapping

**DOI:** 10.3390/s26144604

**Published:** 2026-07-20

**Authors:** Dávid Körmöczi, Gábor Bércesi, György Pillinger, Branislav Šarkan, Péter Kiss

**Affiliations:** 1 Doctoral School of Mechanical Engineering, Hungarian University of Agriculture and Life Sciences, 2100 Gödöllő, Hungary; kormoczi.david@uni-mate.hu; 2 Department of Vehicle Technology, Hungarian University of Agriculture and Life Sciences, 2100 Gödöllő, Hungary; bercesi.gabor@uni-mate.hu (G.B.); pillinger.gyorgy@uni-mate.hu (G.P.); 3 Faculty of Operation and Economics of Transport and Communications, University of Žilina, 8215 Žilina, Slovakia; branislav.sarkan@uniza.sk

**Keywords:** off-road vehicle, lidar, vehicle stability, terrain mapping

## Abstract

The stability of vehicles is one of the most important factors of safety. When assessing the conditions for the stability of the vehicle, on-road and off-road vehicles differ in many aspects, and because of this, specific methods are needed to describe the stability conditions for terrain vehicles. In this paper, the aspects of off-road vehicle stability are assessed, and terrain vehicle-specific stability modeling methods are shown. After that, LiDAR-based terrain mapping technology and the measurement and remote sensing possibilities for the variables affecting stability, both from the vehicle and terrain, are introduced. Field measurements conducted with a small-scale remote-controlled vehicle are presented, the measured and model-predicted stability values are compared, and it is concluded that the theoretical model does describe the actual values accurately enough that the differences can be mitigated by introducing a safety factor. At the end, some further possibilities are proposed for the improvement of the stability modeling method.

## 1. Introduction

Off-road mobility of vehicles is influenced by numerous factors, both from the vehicle and terrain, and modeling methods for terrain vehicles should take into account both of these. While many different mobility models are accessible in the literature, most of them are created by assuming the presence of a driver, who can make manual corrections, and for this reason, these mobility models use a larger scale. However, for unmanned and autonomous vehicles, where no driver is present, the mobility model should be created with an accordingly small scale, so that the vehicle can avoid all obstacles based on the mobility model alone. In this paper, a mobility modeling method is presented, which aims to fulfill these criteria. Existing mobility models have shortcomings in the accuracy and resolution of stability modeling. While the general rule of stability (the pitch and roll angle of the vehicle being below the stability limit) is widely used for vehicle stability analysis, published methods usually make simplifications, not taking into account the continuously changing wheel–soil contact area, the radial stiffness of the wheels, and the suspension displacement. This mobility model assesses the stability, obstacle negotiation capability, and the required traction and grip to determine the mobility through each part of the terrain. After the theoretical basis of the model is shown, laboratory and field experiments are conducted to measure the required parameters of the test vehicle, and a drone-based surveying system is used to create a digital terrain map of the test terrain. At the end, field experiments are conducted on both a natural terrain section and artificial obstacles to experimentally validate the model.

## 2. Materials and Methods

### 2.1. Modeling of the Vehicle Stability

The stability for both on-road and off-road vehicles has two separate factors, and thus two ways to limit the mobility of vehicles on terrain. The roll and pitch stability of the vehicle limits the angle at which the vehicle can travel without a rollover occurring. The base geometrical criterion of a rollover is that the center of mass of the vehicle is positioned outside of the area circumscribed by the contact patch of the wheels [1]. Apart from the geometrical position of the vehicle, this is also affected by the shocks and vibration induced by the movement of the vehicle [2]. For on-road vehicles, a rollover can mostly occur as a result of an impact (tripped rollover), as the vehicles normally travel on close-to-horizontal road surfaces [3]. However, for terrain vehicles, it is not unusual that the vehicles move to terrain surfaces whose slope angle in itself can surpass, or at least be in the range of, the rollover stability limit angle. For this reason, for terrain vehicles, the slope angle is usually a non-negligible factor for rollover stability, and a different approach is required for stability modeling [4,5]. While vibrations can cause a momentary separation of the tire and the ground surface, assessing whether this separation results in actual c stability loss of the vehicle would be not feasible; for this reason, the loss of stability is considered to occur if a wheel loses contact with the ground due to the angle and vibrations of the vehicle [6].

In the following sections, rollover stability modeling will be presented on a 2-dimensional model for the pitch angle; methods used for the roll stability are similar to this.

**Figure 1:**
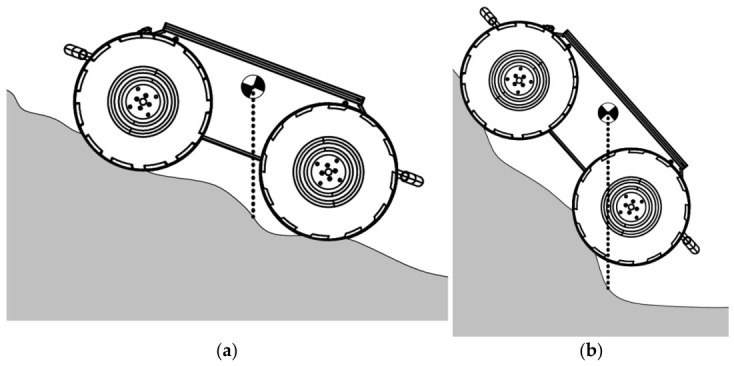
Stability of the vehicle: (**a**) stable position; (**b**) unstable position.

**Figure 2:**
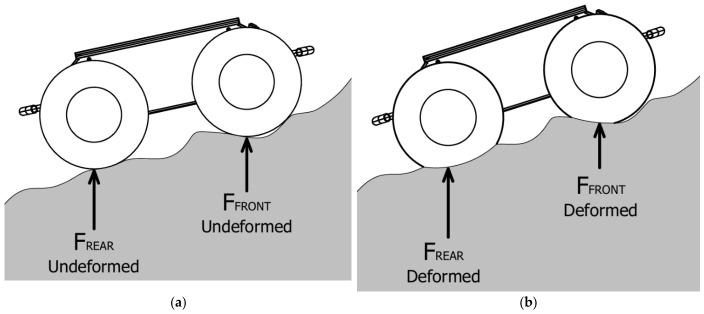
Change in the wheel load distribution: (**a**) undeformed state; (**b**) deformed state.

As shown in Figure 1, the basis for stability is the pitch angle of the vehicle, which is affected by both the terrain surface and the characteristic dimensions of the vehicle. Based on these values, a static pitch angle for the vehicle can be calculated at each point along the path of the vehicle, with basic geometrical methods. This static angle, however, does not represent two important effects: the acceleration due to shocks and vibrations, and the change in the pitch angle due to suspension displacement and wheel–soil deformation characteristics, as it is shown on Figure 2. The deformation of the wheels (and suspension, if present) is an important factor of the stability, since the change in the effective radius of the tire changes the roll/pitch angle of the vehicle. This factor is not negligible for off-road vehicles, which often negotiate terrain obstacles that cause a significant difference between the wheel loads [7]. Both of these displacement values are calculated as a function of the wheel/axle load, and this load is affected by the pitch angle due to the shift in the weight distribution between the wheels [8]. For this reason, an iterative process could be used to calculate the suspension and wheel–soil displacement values. However, to simplify the calculations, an approximation was made to calculate these values based on the wheel load distribution of the undeformed case, considering that the displacement is relatively low compared to the height difference due to the slope angle.

**Figure 3:**
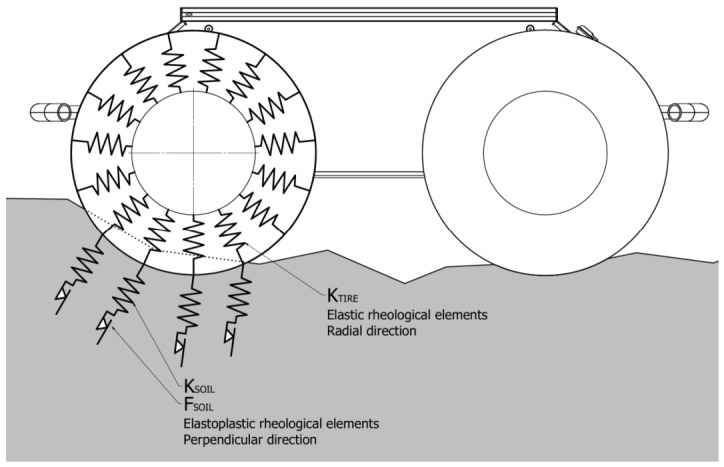
Rheological model of tire–soil deformation.

The tire–soil deformation characteristics are usually described with rheological models, including viscoelastic [9,10] or viscoelastoplastic [11] rheological models. As the test vehicle used in this experiment has a relatively low top speed, it was considered that the viscous part of these models is negligible, and an elastoplastic serial model was used, shown in Figure 3. The parameter identification for this model was carried out by laboratory and field measurements with the test vehicle. As the ground contact surface is not planar, instead of single arithmetical equations or rheological models, which are often used in simplified cases [12], a parallel element model was used, which means that the deformation is characterized by multiple, but independently acting, elements [13].

After the pitch angle is actualized with the suspension displacement and wheel–soil deformation, this static pitch angle is compared to the stability limit angle of the vehicle. For easier comparison, instead of displaying the limit angle in degrees or radians, the actual pitch angle is expressed as a percentage of the limit.

In a static case, this limit would be 100%, or 1, if expressed as a decimal value. This gives a correct assessment of the stability if the vehicle travels at a very slow speed, where dynamic effects are negligible. However, this rollover stability limit needs to be adjusted based on the dynamic vibrations at higher speeds.

The other important aspect of the vehicle safety and stability is the available grip or traction. For terrain vehicles, the available grip is usually limited by the shear strength of the soil, and cannot be expressed as a simple friction coefficient. For this reason, the traction values were obtained using field measurements with the model vehicle. For the safety assessment, the available traction values should be compared to the required traction for safe traverse. This means that the vehicle needs to have enough traction to surpass all resistances and have enough traction to safely conduct emergency braking at any point of the path [14,15]. The motion of the vehicle can be described with the equations

(1) $$F_{a}=F_{t}-R\pm F_{e}$$

(2) $$F_{a}=F_{t}-f\cdot m\cdot g\cdot cos\alpha \pm m\cdot g\cdot sin\alpha$$

where F_a_ is the acceleration force, F_t_ is the traction, R is the rolling resistance, F_e_ is the gravitational resistance, f is the rolling resistance coefficient, g is the gravitational coefficient, m is the vehicle’s mass, and α is the slope angle.

The main resistances to a terrain vehicle are the rolling resistance and the elevational resistance. The elevational resistance is obtained from the pitch angle of the vehicle, and the rolling resistance coefficient is measured. The traction values are obtained from the measurements presented in the next section.

### 2.2. Vehicle Parameter Measurements

For the tests, the unmanned ground vehicle A200 by ClearPath Robotics (Kitchener, ON, Canada) was used. The parameters of the vehicle were measured in laboratory and field conditions. The measured parameters are

- Weight and center of gravity position;
- Moment of inertia;
- Traction on different soil conditions.

#### 2.2.1. Center of Gravity Measurement

**Figure 4:**
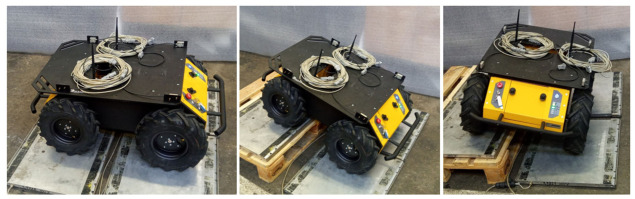
Center of gravity measurement.

The measurement was conducted with a conventional method using wheel scales, tilting the vehicle along the roll and pitch axes [16] as shown in Figure 4. The wheel loads were measured both in horizontal and tilted positions. The measurement results are shown in Table 1.

Based on the measurements, the position of the center of gravity is obtained by the following equations. Longitudinal position is

(3) $$x_{CG}=\frac{L\cdot F_{1}}{F_{1}+F_{2}}$$

where x_CG_ is the longitudinal coordinate of the center of gravity, L is the wheelbase, and F_1_ and F_2_ are the wheel/axle loads in the vertical measurement position.

The transversal position could be calculated with the same method. However, based on the measurements, it was determined that due to the symmetrical build of the vehicle, it is approximately at the symmetry plane of the chassis, so no numerical calculations were needed.

The equation of the vertical position is

(4) $$h_{CG}=\left(x_{CG}-L\frac{F_{3}}{F_{3}+F_{4}}\right)ctg\left(arcsin\frac{H}{L}\right)+R\;\;\;\;\;\;\;\;\;\;\;$$

where h_CG_ is the vertical coordinate of the center of gravity, F_3_ and F_4_ are the wheel/axle loads in the tilted position, H is the platform height, and R is the wheel radius.

**Figure 5:**
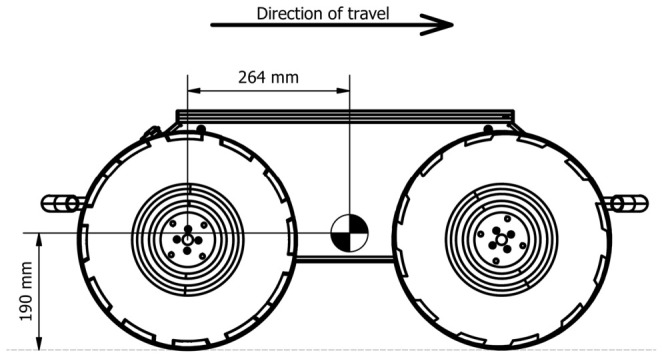
Center of gravity position on test vehicle.

**Figure 6:**
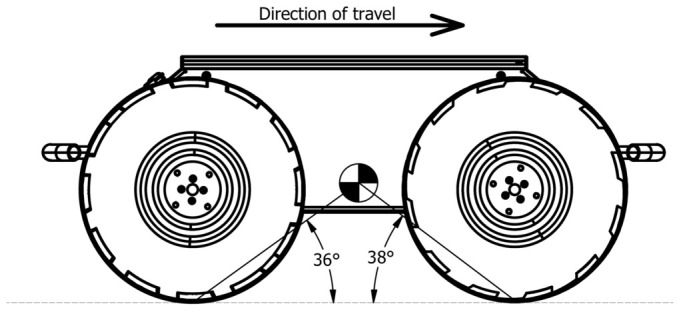
Longitudinal stability limits of test vehicle.

The position of the COG is shown in Figure 5, and the stability limits, represented by static stability angles [17], are in Figure 6.

#### 2.2.2. Moment of Inertia Measurement

**Figure 7:**
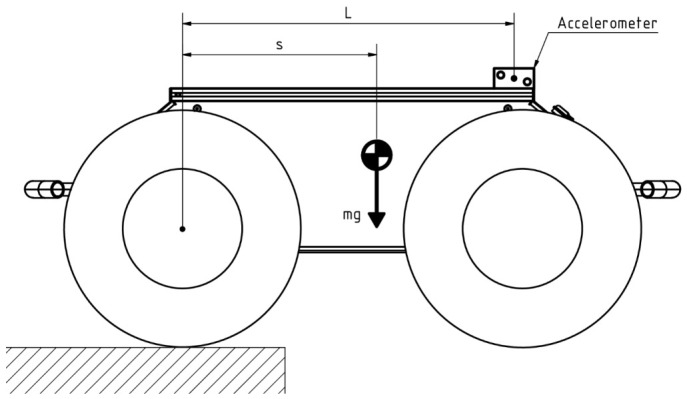
Moment of inertia measurement.

The moment of inertia of a vehicle can be measured with multiple methods. In conventional vehicle dynamic measurements, the moment of inertia is usually measured by bringing the vehicle into oscillation, either using a spring-loaded testbed [18] or the vehicle’s own suspension [19]. However, in this experiment, considering the small scale of the vehicle, the moment of inertia is measured based on methods outside of the vehicle’s technology [20] used for smaller rigid bodies—the gravitational acceleration—while pivoting the vehicle around one of the axes, as shown in Figure 7.

During the measurement, the wheel is disengaged and is in a free rotating state, which means that the center of rotation is on the wheel axis. From the gravitational acceleration, the moment of inertia is derived from the standard equation of the angular acceleration [21], obtained using the following formulas:

(5) $$\sum M=J_{K}\cdot \beta$$

(6) $$mgs=\frac{a}{L}(J+ms^{2})$$

(7) $$J=\frac{mgsL}{a}-ms^{2}$$

where M is the gravitational moment acting on the chassis, J_K_ is the moment of inertia on the wheel axis, β is the angular acceleration, s is the center of gravity position, and J is the moment of inertia on the axis through the center of gravity.

**Figure 8:**
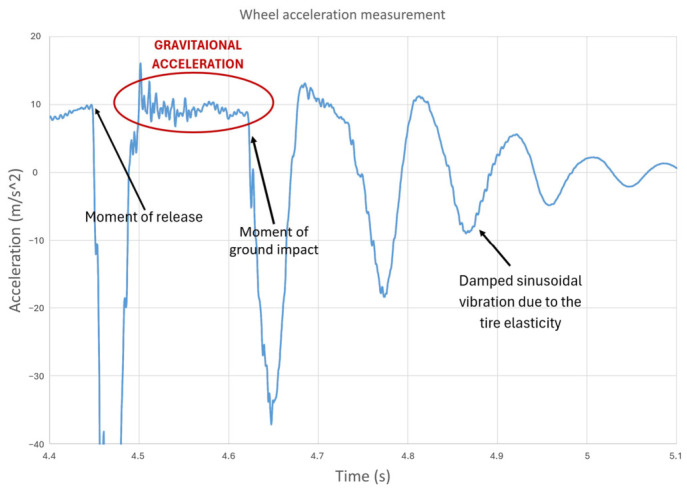
Measured acceleration values.

Figure 8 shows a measured dataset. On the graph, the constant value of the acceleration due to gravity can be clearly seen.

In total, 6 measurements were conducted. For each measurement, 2 accelerometers of the type KAS-903 by Kelag (Schwerzenbach, Switzerland) were used. The average of these 2 values was considered for the calculations; these values can be seen in Table 2.

#### 2.2.3. Traction Measurement

**Figure 9:**
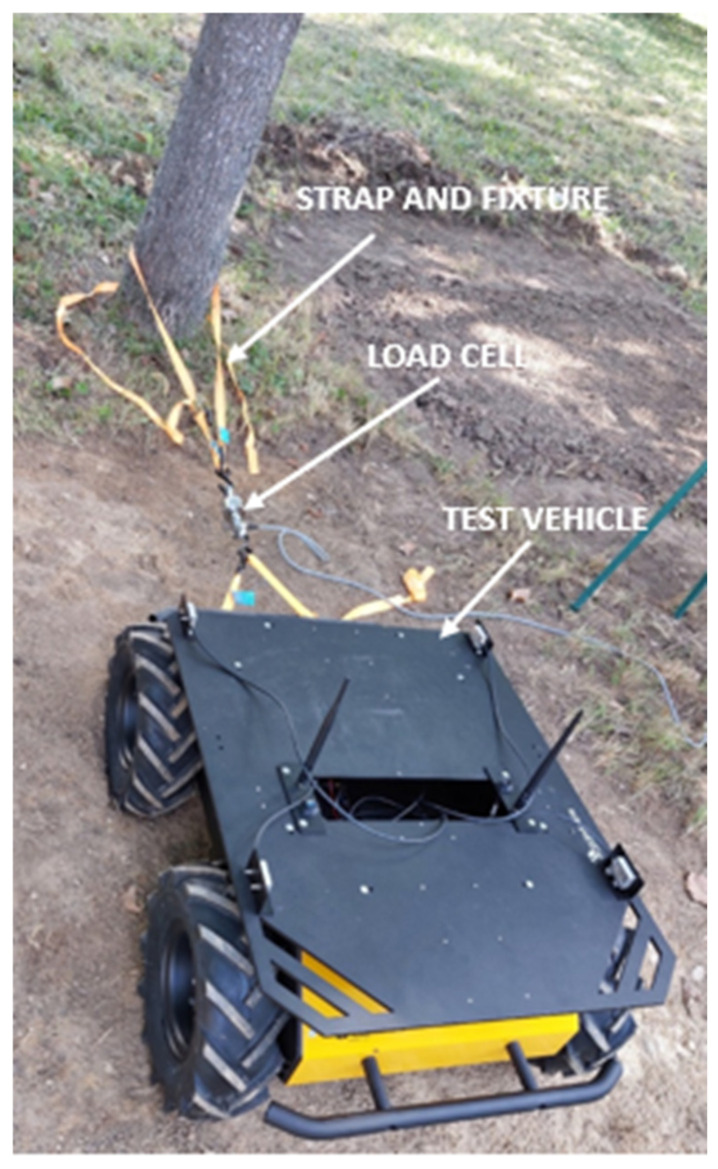
Traction measurement in fixed position.

**Figure 10:**
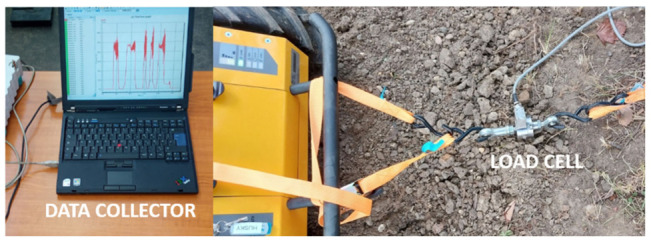
Measurement setup for traction measurement.

The traction of the vehicle was measured under static conditions. The traction was measured using a force inducer through a towing strap. The measurements were conducted on soils with different densities, on soil covered with vegetation, and with different payloads on the vehicle. Changing the tire pressure during the measurements was also considered, but based on the pilot measurement, it was assumed that its effect on the traction is negligible. The measurement setup is seen in Figure 9 and Figure 10.

**Figure 11:**
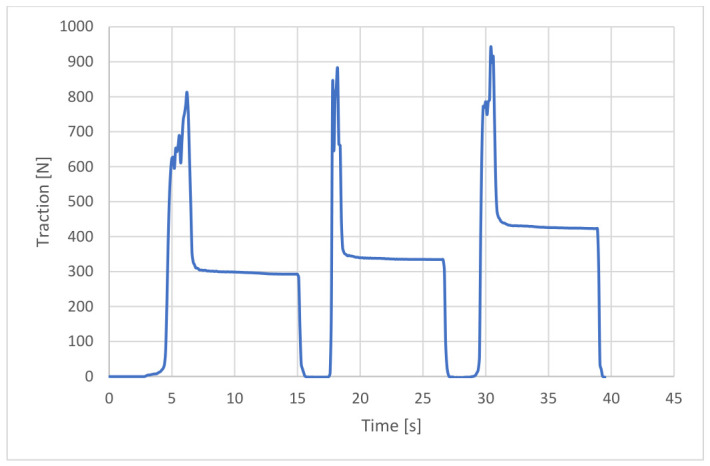
Results of traction measurement (20 kg load, loose soil).

With each load and soil type, the traction was measured 3 times, as seen in Figure 11. On the graph, the intervals of static and dynamic friction can be seen. The difference between the local maxima and minima is explained by the friction coefficient changing as a function of the relative speed of the frictional surfaces (the layer of the tire–soil contact, and the internal sheared surfaces inside the soil). This relative speed is, in this case, proportional to the wheel slip. The traction values, especially near the local maxima, are also influenced by the slight changes in the vertical load on the wheels, caused by the oscillation induced by the spinning wheels. For the available traction value, the average of the dynamic frictional intervals was calculated.

### 2.3. Setup for Validation Measurements

**Figure 12:**
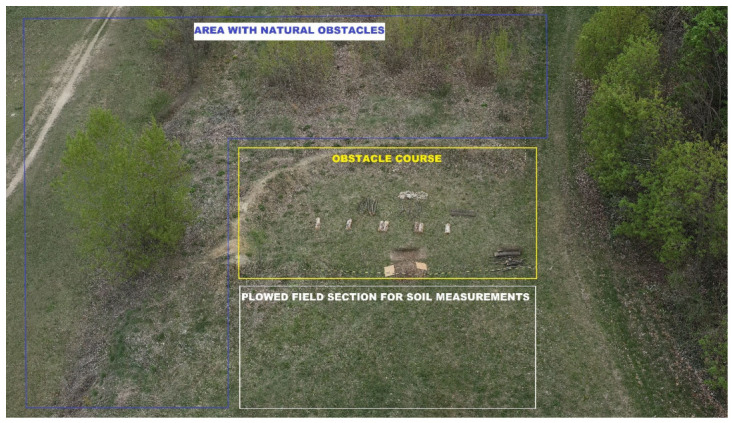
Aerial view of test area.

**Figure 13:**
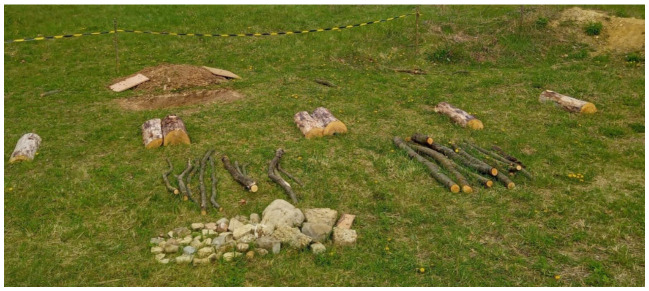
Obstacle course on the test area.

The experimental measurements were conducted in a test field at the Hungarian University of Agriculture. On the terrain, a set of obstacles fitting the scale of the vehicle was built, which is shown in Figure 12 and Figure 13.

**Figure 14:**
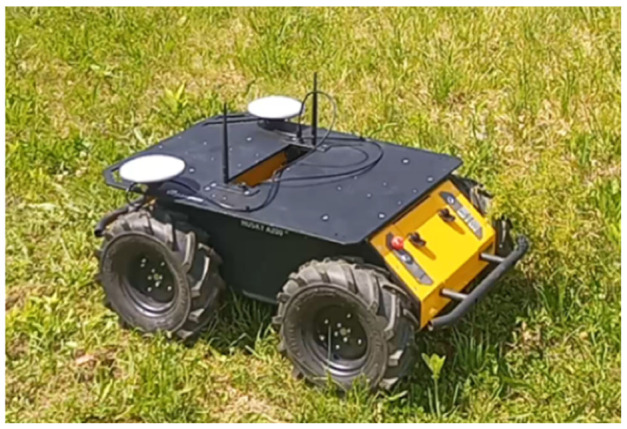
Test vehicle outfitted with sensor array.

For the tests, the vehicle was outfitted with an array of sensors, which is shown in Figure 14.

The position of the vehicle was obtained using a stereo RTK/GNSS system, which gives both the position and orientation of the vehicle. The positional data was collected with a resolution of 10^−7^° (approximately 1 cm) on both the longitude and latitude coordinates independently, and the accuracy of the measurement was between 3 and 5 cm. The orientation was also measured using an inertial measurement unit (IMU). During the tests, the traction (obtained from the torque of the motors) was logged by the vehicle’s control unit as a function of time. These values were correlated to the position of the vehicle based on the timestamps. The acceleration at each wheel was measured using the same measurement sensors introduced in the “Moment of inertia measurement” Section 2.2.2.

### 2.4. LiDAR-Based Terrain Mapping

For terrain mapping purposes, the most applied method is unmanned aerial vehicle (UAV)-based remote sensing. Two sensing techniques are used by devices mounted on UAVs. Photogrammetry relies on capturing high-resolution images (usually RGB images) with low-distortion optical cameras with appropriate overlap between neighboring images. Knowing the precise geolocation of the UAV, software identifies matching points between images taken at different locations and determines their spatial coordinates through triangulation. As a result, the software tool can generate a digital terrain model in the form of a point cloud from the series of plain images. This method is quick, does not require special sensory tools besides a good-quality camera, can result in a relatively good-resolution point cloud and directly provides RGB color information assigned to each point. However, it has drawbacks due to the influencing effect of illumination of the surface and shading effects and has limited accuracy of 3–6 cm of uncertainty in estimating point coordinates. LiDAR provides an alternative UAV-based surveying method. As an active sensor, it determines the distance to surface points by measuring the time of flight of emitted laser pulses. LiDAR can rapidly generate dense point clouds comparable to those from photogrammetry, and can also record multiple returns from a single pulse, enabling partial detection of vegetation-covered surfaces.

Cooperative use of autonomous and remote-controlled land and air vehicles is an often-studied field in vehicle control and dynamics. In this research, the drone system that surveyed the test terrain was pre-programmed to fly a pattern over the entire test area; however, similar terrain mapping in real time could be conducted by the UAV flying above the ground vehicle and surveying the terrain in real time [22].

Accurate positioning and orientation of the UAV and its sensors are essential for both photogrammetric and LiDAR-based surveying. These data are usually acquired from centimeter-accuracy RTK-corrected GNSS services combined with acceleration, angular velocity and geomagnetic field strength measurement from onboard inertial measuring units (IMUs). The absolute accuracy of positioning the point cloud can be improved further by applying ground control points of known positions around the surveying area to use as references for calibration.

**Figure 15:**
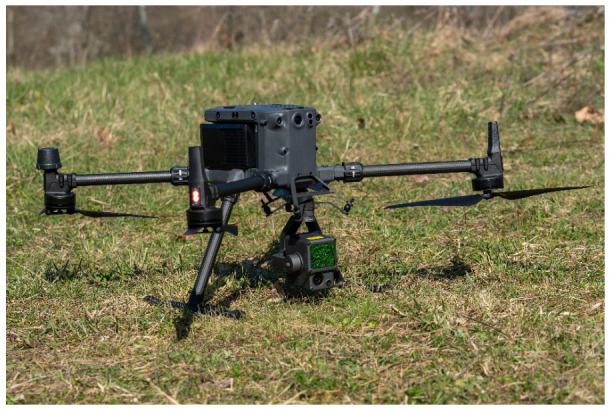
Drone-borne measurement system.

In surveying the MATE test field for mobility mapping purposes, a DJI (Shenzhen, China) Matrice 350 RTK quadcopter (shown on Figure 15) UAV equipped with a DJI Zenmuse L1 LiDAR scanner was applied for airborne terrain mapping. The main specifications of the LiDAR are the following:

- Maximum sensing range 450 m.
- Single- or triple-return detection modes.
- Point rate: 240,000 points/s (single) and 480,000 point/s (triple).
- Standard uncertainty @ 50 m: 10 cm (horizontal) and 5 cm (vertical).
- Real-time depth-based, distance-based, reflectance-based or visible camera RGB camera-based coloring modes.
- Field of view: 70.4° (horizontal) × 77.2° (vertical) with non-repetitive scanning pattern or 70.4° (horizontal) × 4.5° (vertical) with repetitive scanning pattern.
- Supplementary RGB mapping camera resolution: 20 MP (5472 × 3078 or 4864 × 3648 or 5472 × 3648 depending on aspect ratio setting).

Measurement setup was supplemented with an Emlid Reach RS2+-type GNSS receiver operating as a local RTK base station to provide a correction signal for accurate UAV positioning. The base station was set up on a previously surveyed ground reference point close to the testing area and had access to Hungarian network RTK service MAXI-NET 2.0 to improve absolute positioning. The UAV flew an ortho-mapping mission covering approximately 15,000 m^2^. Key planning parameters were

- Flight altitude: 38 m, relative to take-off point (AGL).
- Elevation optimization enabled.
- Ground speed: 3.6 m/s.
- Point density: 693 points/m^2^.
- Triple-return mode.
- RGB camera ground sampling distance (GSD): 1.04 cm/px.
- Side overlap ratio—LiDAR: 20%;—RGB camera: 38%.
- Frontal overlap ratio (RGB camera): 70%.

**Figure 16:**
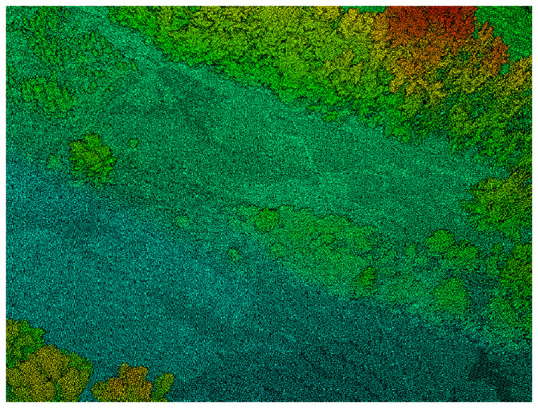
Point cloud obtained by LiDAR mapping.

**Figure 17:**
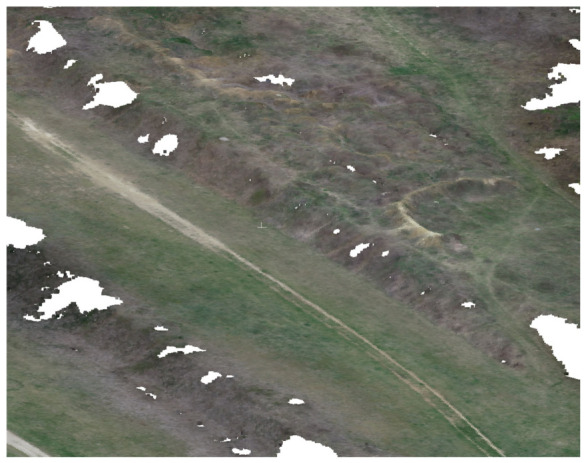
Rasterized digital terrain model of the test area.

The selected resolution is determined by multiple factors. Naturally, a higher resolution is generally favorable. However, since the accuracy of the LiDAR is limited—usually in the range of a few centimeters—there is a reasonable limit where the increase in the spatial resolution would only give diminishing returns [23]. The practically applicable resolution is also limited by both the surveying time and the available computational time for evaluating and manipulating the obtained data [24]. For these reasons, the usually applied point density is in the magnitude of ten points per square meter [25]. In our specific research, however, since the surveyed area was considered small and there was no time requirement to process the data, we opted for a relatively high resolution of approx. 700 points per square meter. The recorded point cloud (Shown on Figure 16) was then processed in DJI Terra 5.1.1 software. The raw point cloud data were imported to the software where—by applying the proper settings—a raster grid was created with a 10 cm vertical sampling resolution, which can be seen on Figure 17. The raster resolution was chosen based on the consideration that for the earlier explained rheological modeling of the tire–terrain interaction, multiple (preferably at least 3–5) data points are required in the area characterized by the wheel dimensions.

## 3. Results

**Figure 18:**
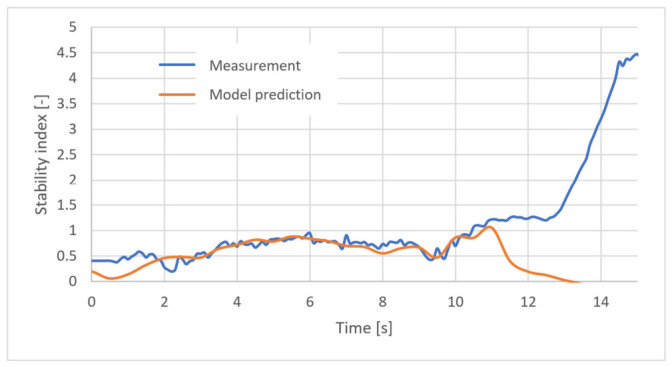
Results of the validation measurement.

During the measurement, data was collected at approximately 12,000 data points during the travel of the vehicle. The test was conducted in one continuous route on the entire test area, and the measurement was reset only when a rollover or other failure stopped the travel of the vehicle. In the following section, the results are shown for the vehicle traversing on a slope-type obstacle. In Figure 18, the comparison of the predicted and measured stability index is shown. As discussed earlier, by using this decimal value, the limit of vehicle stability is 1. As can be seen on the graph, in the range of the stability index below 1, the prediction accurately represents the measured values. We can see a significant difference at the beginning, approximately between 0 and 2 s. This can be explained by the initial acceleration of the vehicle from a static position, as this large longitudinal acceleration influences the inertial measurement.

**Figure 19:**
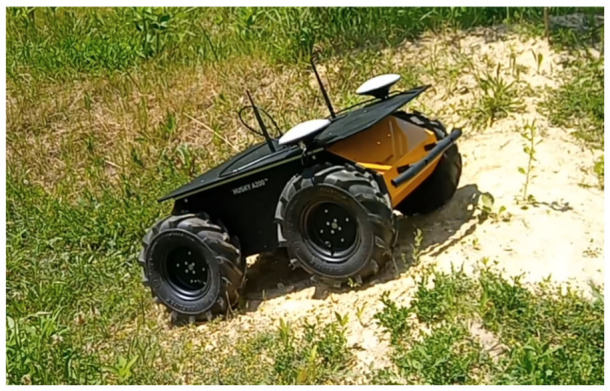
Stable position of the vehicle during the tests.

**Figure 20:**
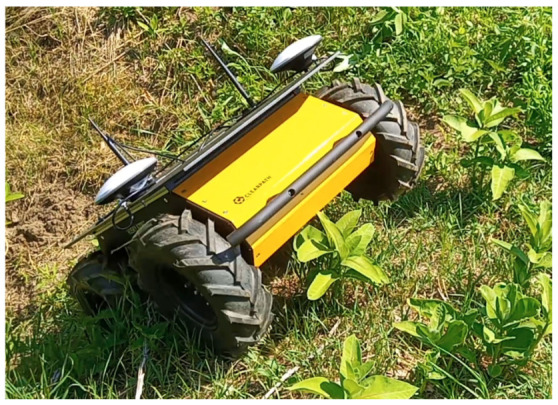
Vehicle at the point of stability loss.

The loss of stability can be seen in the graph, when the value of the stability index surpasses the value 1 at approximately 11 s. This is the point of stability loss. Before reaching the point of of the stability limit, the vehicle is in a stable position, similarly as shown on Figure 19. At the point of the stability loss, which is pictured in Figure 20. during the field measurements, the vehicle rolls over uncontrollably, which explains why the predicted and measured values after this point diverge.

## 4. Discussion

**Figure 21:**
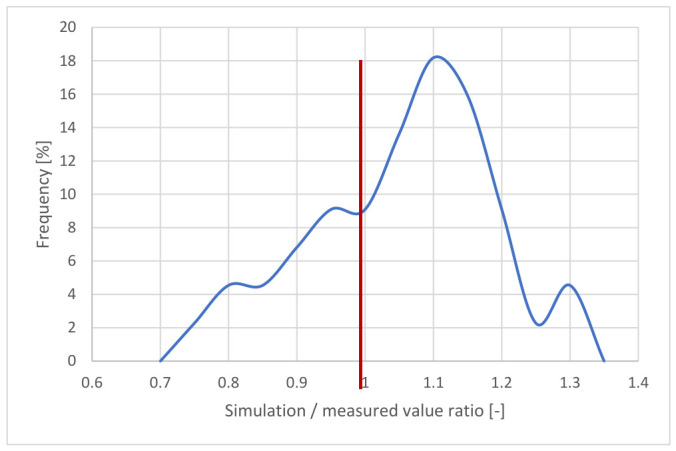
Error histogram of the stability model.

The accuracy of the stability prediction can be described by conventional statistic methods. However, besides the mathematical accuracy, the differentiation between false negatives and positives is very important in this case. A false positive error means that the vehicle is predicted to be stable and safe when traversing through an obstacle according to the model, while in reality a loss of stability will occur, which can lead to material, and even bodily, harm. On the contrary, a false negative prediction only means that the vehicle will be restricted from a route that could be physically traversable, which could make route planning and operation suboptimal, but does not negatively affect safety. The error histogram of the model is shown in Figure 21. It can be seen that the model is biased towards the side of false negatives, which is favorable from a safety perspective. False positive predictions still occur, but these can be mitigated by introducing a safety factor, which shifts all errors into the false negative field, although this also introduces the aforementioned suboptimality.

The mobility modeling method and field measurements conducted in this research are an extended version of the authors’ earlier research, where the initial results were briefly discussed. Based on the results, it can be concluded that the off-road vehicle-specific theoretical model of stability introduced in the research does predict the actual values measured on terrain obstacles accurately enough that the difference can be mitigated by introducing a safety factor. This comparison of model accuracy also proves the practical applicability of this novel stability modeling method for terrain mobility assessment of off-road vehicles. The accuracy of the results could be further detailed by only examining this ratio of the model-predicted and measured values near the limit of stability.

## Figures and Tables

**Table 1:** Wheel load measurement results.

Axle Load (kg)		Wheel Lift Height (mm)	
Front	Rear	Front	Rear
26.1	24.5	0	0
35.6	15	0	155
35	15.6	250	0

**Table 2:** Moment of inertia measurement results.

Measurement	a (m/s^2^)	J (kgm^2^)
1	9.39	3.67
2	9.33	3.71
3	9.24	3.78
4	9.20	3.81
5	9.35	3.69
6	9.45	3.62

## Data Availability

The original contributions presented in this study are included in the article. Further inquiries can be directed to the corresponding author.
